# Anti-Inflammatory Effects of Polyphenols in Brain Microvascular Endothelial Cells Stimulated with TNF-α

**DOI:** 10.3390/ijms27031316

**Published:** 2026-01-28

**Authors:** Joanna Czpakowska, Andrzej Glabinski, Piotr Szpakowski

**Affiliations:** Department of Neurology and Stroke, Medical University of Lodz, Zeromskiego 113 Street, 90-549 Lodz, Poland; joanna.czpakowska@umed.lodz.pl

**Keywords:** polyphenols, inflammation, chrysin, myricetin, curcumin, resveratrol, brain endothelial cells, blood–brain barrier, neuroinflammation, neurodegeneration

## Abstract

The blood–brain barrier (BBB) is a structure that regulates the exchange of substances between the peripheral circulation and the central nervous system (CNS), thereby protecting this environment. An increase in BBB permeability may lead to the influx of inflammatory cells, resulting in neuroinflammation and neurodegeneration. The integrity of the BBB is maintained due to the specific properties of brain endothelial cells. Considering the importance of brain endothelial cells in the BBB during inflammatory processes, these cells may be a target for anti-inflammatory agents. Polyphenols are substances exhibiting the ability to decrease inflammation; therefore, in our research, we aimed to examine their effectiveness in a brain endothelial cell culture stimulated with the pro-inflammatory cytokine TNF-α. The tested polyphenols were myricetin, chrysin, resveratrol, and curcumin. ELISA tests revealed that myricetin and chrysin decreased the concentrations of the pro-inflammatory cytokines IL-1ß, IL-6, and IL-8 secreted by brain endothelial cells. The results of flow cytometry indicate that chrysin and resveratrol are the most potent in downregulating the expression of *VCAM-1* on the surface of brain endothelial cells. The obtained results confirm the anti-inflammatory potential of polyphenols in brain endothelial cells. The selected polyphenols also contribute to increasing brain endothelial cell viability and act as antioxidants.

## 1. Introduction

Brain endothelial cells constitute the main component of the BBB, which plays a crucial role in the communication between the CNS and the circulatory system [1]. The proper functioning of brain endothelial cells, along with perivascular cells, astrocytes, microglia, and neuronal endings, which form the neurovascular unit (NVU), is essential in maintaining homeostasis in the CNS [2,3,4]. Brain endothelial cells contribute to the low permeability of the BBB due to their specific properties. The presence of tight junction (TJ) complexes on brain endothelial cells, formed by occludin, claudin-5, and junctional adhesion molecules, supported by VE-cadherin and E-cadherin, limits paracellular transport [5,6]. Transcellular passage is also restricted in brain endothelial cells [6,7]. Taken together, brain endothelial cells contribute to the activity of the BBB, which is able to prevent inflammation, inhibit toxins from entering the CNS, maintain ion balance, and correct signaling.

The improper functioning of the BBB can contribute to the emergence and progression of neurodegenerative and neuroinflammatory processes, leading to neurological dysfunctions such as multiple sclerosis (MS), Parkinson’s disease (PD), traumatic brain injury (TBI), and stroke, which highlights the importance of brain endothelial cells [2,8]. In the case of MS, the loss of BBB tightness, which is mainly based on TJs of brain endothelial cells, leads to the influx of immune cells. This process triggers inflammation and leakage of plasma proteins, such as fibrinogen, which results in their accumulation in demyelinating lesions [9,10,11]. Similar processes are observed in PD, where the entrance of α-synuclein precedes the secretion of inflammatory agents by NVU cells through the impaired BBB [12]. TBI can also be the cause of BBB damage, which can spread more intensively due to inflammatory responses after activation of microglia and astrocytes by leaking albumin [13]. The disruption of the BBB that takes place after a stroke is driven by oxidative stress. At the initial stage, reactive oxygen species (ROS) affect TJs and the cytoskeleton of brain endothelial cells. Next, metalloproteinases (MMPs) degrade TJs. As a consequence of BBB damage, cerebral edema emerges. Brain endothelial cells take part in ionic edema directly because their ion channels allow increased passage of Na^+^, Cl^−^, and water. To summarize, BBB integrity is based on the proper functioning of brain endothelial cells, especially of their TJs. Neuroinflammatory and neurodegenerative processes contribute to BBB dysfunction; therefore, brain endothelial cells constitute a promising target for future therapeutic approaches [10,14].

One of the candidates demonstrating anti-inflammatory properties is polyphenols. The group of phenolic compounds is divided into various sub-groups such as flavonoids, stilbenes, phenolic acids, and lignans. The division is based on similarities in their primary structures [15,16]. Polyphenols are widely available in fruits, herbs, and spices, which means that the human diet provides many sources of them. The role of these compounds in plants is not fully understood and may be implicated in defense against ultraviolet radiation and hostile microbial agents. In addition to these functions, polyphenols act as antioxidants by inhibiting ROS formation. Polyphenols can suppress enzymes that catalyze ROS synthesis, eliminate them directly, or enhance defense mechanisms. The reduction of ROS by polyphenols contributes to the constriction of tissue injuries, which can otherwise lead to inflammation. Additionally, the involvement of polyphenols in the inhibition of inflammatory signaling pathways and in the activity of inflammatory cells and enzymes engaged, for instance, in arachidonic acid metabolism, proves their anti-inflammatory effect [17,18,19].

## 2. Results

### 2.1. Stimulation of HBEC-5i Cells with TNF-α Resulted in the Increased Secretion of Pro-Inflammatory Cytokines

**Figure 1:**
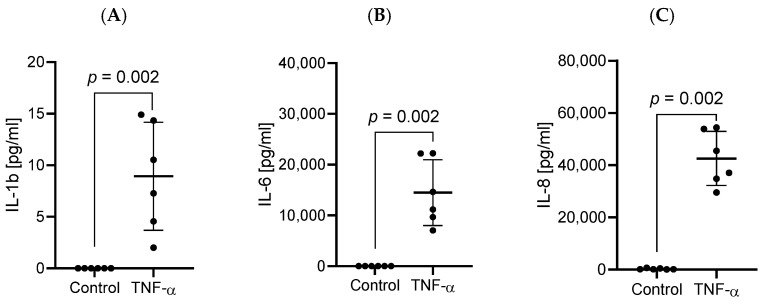
Effect of 48 h TNF-α exposure on IL-1β (**A**), IL-6 (**B**), and IL-8 (**C**) production in HBEC-5i cultures. Data are presented as individual values (*n* = 6 per group) together with mean ± SD. Statistical comparison between groups was performed using the Mann–Whitney U test to confirm the biological activity of TNF-α.

In response to the stimulation of HBEC-5i cells with TNF-α, the secretion of the pro-inflammatory cytokines IL-1ß (*p* = 0.0022), IL-6 (*p* = 0.002), and IL-8 (*p* = 0.002) was intensified in comparison to the control group with medium alone where these cytokines were not detected (Figure 1).

### 2.2. Treatment of HBEC-5i Cells with Polyphenols Reduced the Concentration of Secreted Pro-Inflammatory Cytokines

**Figure 2:**
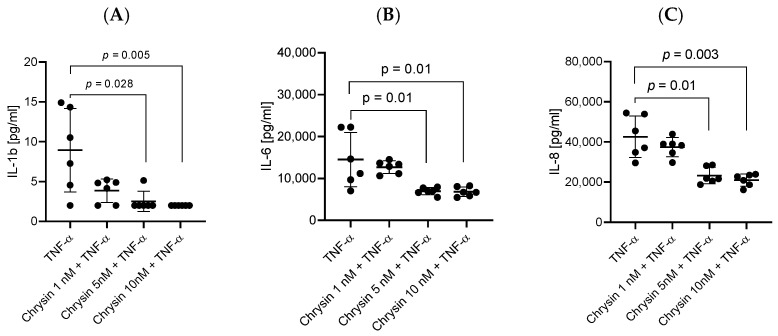
Impact of chrysin (1 nM, 5 nM, and 10 nM) on cytokine production in HBEC-5i cells following 48 h TNF-α stimulation. Production of IL-1β (**A**), IL-6 (**B**), and IL-8 (**C**) was measured in HBEC-5i cultures. Data are presented as individual values (*n* = 6 per group) together with mean ± SD. Statistical comparisons between groups were performed using the Kruskal–Wallis test followed by Dunn’s post-hoc analysis.

**Figure 3:**
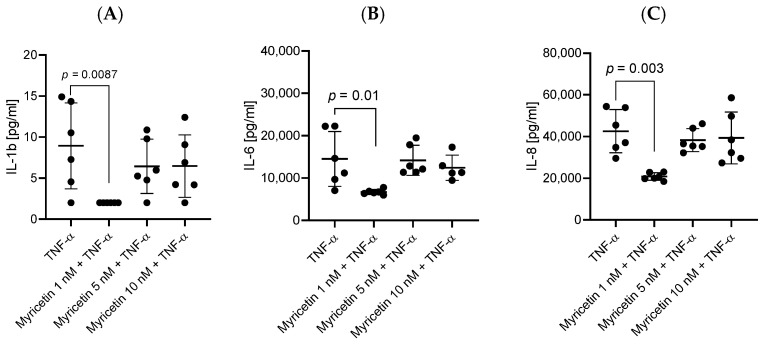
Impact of myricetin (1 nM, 5 nM, and 10 nM) on cytokine production in HBEC-5i cells following 48 h TNF-α stimulation. Production of IL-1β (**A**), IL-6 (**B**), and IL-8 (**C**) was measured in HBEC-5i cultures. Data are presented as individual values (*n* = 6 per group) together with mean ± SD. Statistical comparisons between groups were performed using the Kruskal–Wallis test followed by Dunn’s post-hoc analysis.

**Figure 4:**
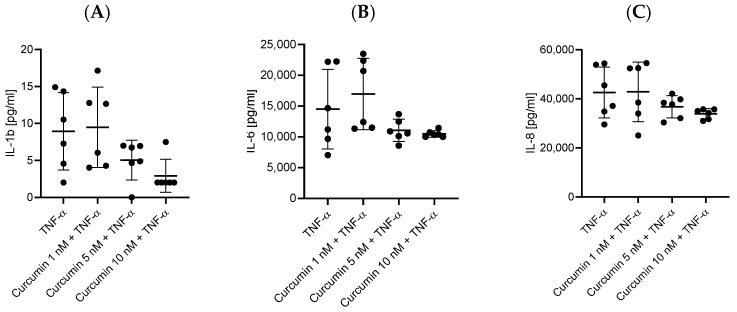
Impact of curcumin (1 nM, 5 nM, and 10 nM) on cytokine production in HBEC-5i cells following 48 h TNF-α stimulation. Production of IL-1β (**A**), IL-6 (**B**), and IL-8 (**C**) was measured in HBEC-5i cultures. Data are presented as individual values (*n* = 6 per group) together with mean ± SD. Statistical comparisons between groups were performed using the Kruskal–Wallis test followed by Dunn’s post-hoc analysis.

**Figure 5:**
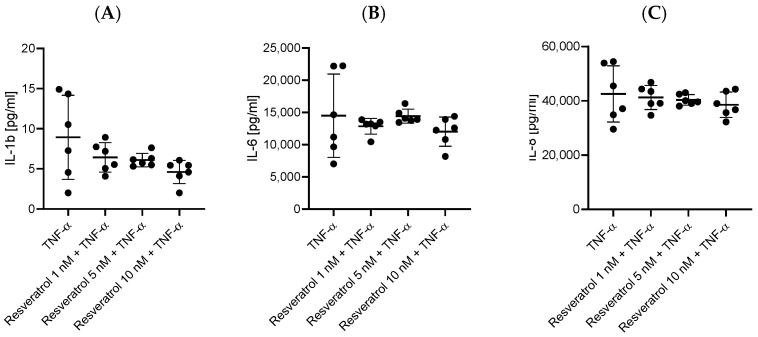
Impact of resveratrol (1 nM, 5 nM, and 10 nM) on cytokine production in HBEC-5i cells following 48 h TNF-α stimulation. Production of IL-1β (**A**), IL-6 (**B**), and IL-8 (**C**) was measured in HBEC-5i cultures. Data are presented as individual values (*n* = 6 per group) together with mean ± SD. Statistical comparisons between groups were performed using the Kruskal–Wallis test followed by Dunn’s post-hoc analysis.

HBEC-5i cells stimulated simultaneously with TNF-α and one of the tested polyphenols (chrysin, myricetin, curcumin, and resveratrol), at 1 nM for myricetin and 5 or 10 nM for the others, showed a decrease in the levels of secreted pro-inflammatory cytokines compared to stimulation with only TNF-α. The most effective concentrations lowering the levels of IL-1ß, IL-6, and IL-8 turned out to be 10 nM for chrysin, curcumin, and resveratrol and 1 nM for myricetin. Statistically significant results in downregulating the expression of IL-1ß, IL-6, and IL-8 are assigned to chrysin (*p* = 0.005, *p* = 0.01, and *p* = 0.0033, respectively) (Figure 2) and myricetin (*p* = 0.0087, *p* = 0.01, *p* = 0.0033) (Figure 3). Notably, curcumin (Figure 4) and resveratrol (Figure 5) also reduced concentrations of secreted cytokines; however, their effects were not statistically significant.

### 2.3. Exposure of HBEC-5i Cells to TNF-α Led to the Upregulation of CD106 (VCAM-1) Expression

**Figure 6:**
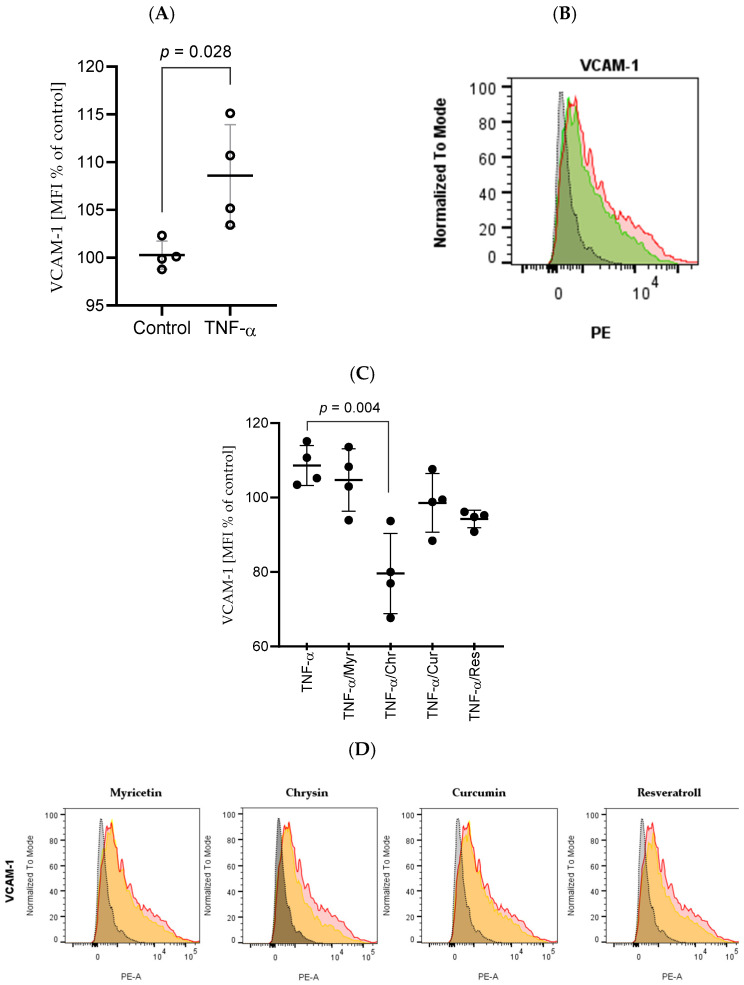
Impact of the tested polyphenols on *VCAM-1* surface expression on HBEC-5i cells under different culture conditions. (**A**) Effect of TNF-α stimulation on *VCAM-1* expression in brain microvascular endothelial cells; data from four independent measurements, normalized to the control (cells cultured in medium). (**B**) Representative histograms: gray histogram with a dotted contour line—FMO control; green—culture medium; red—TNF-α stimulation. (**C**) Effects of the tested polyphenols on TNF-α-induced *VCAM-1* expression; statistical evaluation performed using Kruskal–Wallis test followed by Dunn’s post-hoc analysis. (**D**) Representative histograms (gray—FMO control, red—TNF-α, orange—polyphenol + TNF-α). The biological effect of TNF-α on *VCAM-1* expression in HBEC-5i cells was confirmed using the Mann–Whitney U test. Graphs show median fluorescence intensities of individual samples normalized to the control group (dots) together with mean ± SD.

Flow cytometry was used to measure the expression of CD106 on the surface of HBEC-5i cells. Stimulation of HBEC-5i cells with TNF-α resulted in an increased level of CD106 in comparison to unstimulated cells (*p* = 0.028) (Figure 6).

### 2.4. Chrysin, Myricetin, Curcumin, and Resveratrol Caused Downregulation of CD106 Expression

Analysis of flow cytometry results from HBECs stimulated with TNF-α and polyphenols showed weaker CD106 upregulation in response to TNF-α in the presence of chrysin (*p* = 0.028) and resveratrol (*p* = 0.028). Stimulation of cells with curcumin and myricetin did not result in a statistically significant downregulation of CD106.

### 2.5. The Application of Polyphenols Confirmed Their Antioxidative Properties and Resulted in the Improvement of Cerebral Microvascular Endothelial Cell Viability

**Figure 7:**
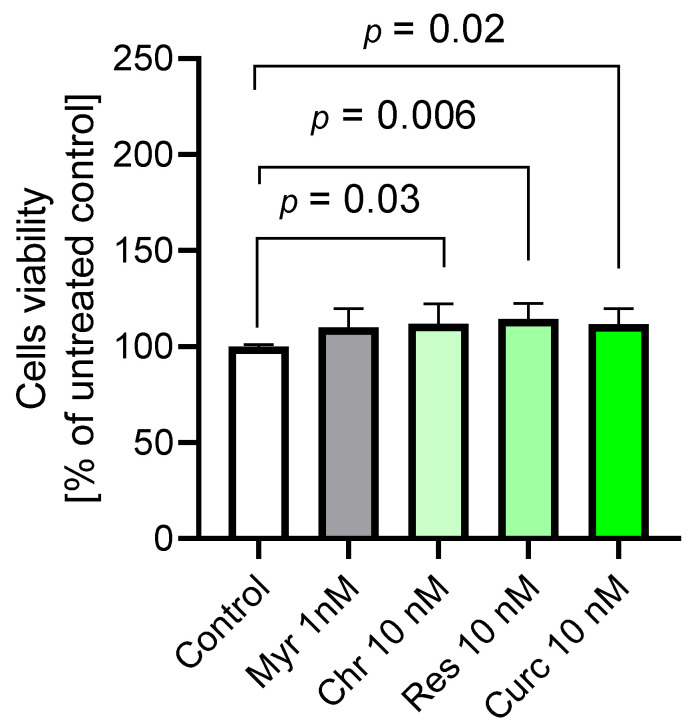
Impact of the tested polyphenols on HBEC-5i cell viability assessed by the EZMTT assay. Cell viability is expressed as the percentage of absorbance values normalized to the mean value of the control group (cells cultured in medium). Data are presented as mean ± SD, *n* = 6. Statistical comparisons between groups were performed using the Kruskal–Wallis test followed by Dunn’s post-hoc analysis.

**Figure 8:**
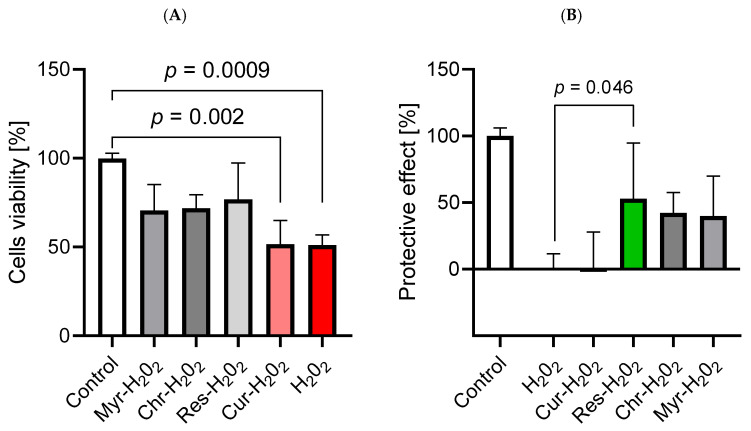
Protective effect of tested polyphenols on H_2_O_2_-induced oxidative stress in human brain endothelial cells. (**A**) Cell viability following co-treatment with H_2_O_2_ and selected polyphenols. Viability is expressed as a % relative to the untreated control (medium), set as 100%. (**B**) Calculated protective effect of the tested polyphenols. The H_2_O_2_-treated group was set as 0% protection and the untreated control (medium) as 100%. Statistical analysis was performed using the Kruskal–Wallis test followed by Dunn’s post-hoc test. Comparisons were made against the H_2_O_2_ group to assess the significance of protective effects. A *p*-value < 0.05 was considered statistically significant. Data are presented as mean ± SD, (*n* = 6 per group).

The obtained results indicated that chrysin, resveratrol, and curcumin were involved in improving HBEC-5i cell viability (*p* = 0.03, *p* = 0.006, *p* = 0.02, respectively), which may be attributed to an increased mitochondrial metabolic rate or enhanced cellular proliferation. Myricetin did not show this effect. Tested compounds at the indicated concentrations did not reduce cell viability, but increased it, compared to the untreated control (medium), indicating a lack of intrinsic cytotoxicity (Figure 7). Simultaneously, these polyphenols acted as oxidants in groups of cells treated with H_2_O_2_. The viability of cells treated with H_2_O_2_ was significantly lower than that in the group with medium alone (*p* = 0.0009). Simultaneous treatment of cells with polyphenol and H_2_O_2_ resulted in increased viability. Only curcumin did not show the antioxidant effect (Figure 8A). The protective effect of polyphenols was the most visible in the case of resveratrol, which most effectively improved viability compared to the H_2_O_2_ group (*p* = 0.046) (Figure 8B).

## 3. Discussion

In the context of polyphenols’ application in the prevention and treatment of neurodegenerative and neuroinflammatory diseases, it is essential to analyze the molecular mechanisms underlying their anti-inflammatory, immunomodulatory, and antioxidant activities. Polyphenols modulate the immune system in many diverse ways. They can influence the production of proinflammatory cytokines and the activity of immune cells, and they can inhibit the expression of proinflammatory genes. Additionally, polyphenols affect many signaling pathways associated with immunity, such as nuclear factor kappa-light-chain-enhancer of activated B cells (NF-κB) and mitogen-activated protein kinase (MAPK). Polyphenols also contribute to ROS elimination by suppressing enzymes involved in their synthesis, such as NADPH oxidase (NOX), and by increasing the production of antioxidant enzymes, for instance, superoxide dismutase (SOD). This antioxidant activity reduces inflammation [20,21].

There are a few promising polyphenols worth attention for their anti-inflammatory activity. One of them is chrysin, a flavone from the flavonoid family [22]. Flavonoids constitute the most abundant group of polyphenols in our diet, with an average intake of 50–800 mg/day. In addition to their anti-inflammatory activity, they have antioxidant, neuroprotective, antiviral, cardioprotective, and anticarcinogenic properties [22,23]. Similar to other polyphenols, chrysin exhibits low bioavailability (in the case of oral consumption: 0.003–0.02%) and poor solubility. Additionally, it is metabolized rapidly and quickly eliminated. Therefore, to fully exploit the potential of chrysin and other polyphenols, strategies that overcome these restrictions must be developed [22,23,24].

The anti-inflammatory activity of chrysin is based on its modulation of key immunomodulatory mechanisms. Chrysin can decrease the levels of pro-inflammatory enzymes, including myeloperoxidase (MPO), cyclooxygenase-2 (COX-2), and inducible nitric oxide synthase (iNOS), by activating the peroxisome proliferator-activated receptor gamma (PPAR-γ). Inhibiting the NF-κB signaling pathway is another way of lowering the expression of iNOS and COX-2 by chrysin [25]. Additionally, suppressed by chrysin, NF-κB is not able to induce the production of pro-inflammatory cytokines such as IL-1β, IL-6, and IL-8 [26]. MAPK is another signaling pathway that can be influenced by chrysin. Among all kinases belonging to the MAPK family, which are extracellular signal-regulated kinase (ERK), c-Jun N-terminal kinase (JNK), and p38, this polyphenol selectively inhibits JNK by reducing its phosphorylation. As a result, the secretion of proinflammatory cytokines is decreased [27].

Another noteworthy member of the flavonoid family and the flavonol sub-group is myricetin. This polyphenol exhibits properties similar to those of other flavonoids, including anti-inflammatory and antioxidant activities. The distinguishing feature of myricetin is its high effectiveness at low doses in the elimination of free radicals [28,29]. The increased amount of hydroxyl groups in comparison to other flavonoids (three groups on ring B) in the myricetin chemical structure is thought to be responsible for its antioxidant properties [28,30]. Contrarily, some studies indicated that myricetin can also act in a pro-oxidative way by reducing oxygen to ROS and iron (III) to iron (II) [31].

Myricetin, similarly to chrysin, can inhibit the NF-κB pathway and downregulate iNOS and COX-2 [32,33]. In terms of the MAPK pathway, myricetin does not act selectively but inhibits all of its kinases (ERK, JNK, and p38) [32]. Myricetin, by inhibiting toll-like receptor 2 (TLR2), suppresses the activity of the mTOR signaling pathway, which contributes to lowered production of pro-inflammatory cytokines such as IL-6 and IL-1β [34]. In the context of CNS inflammatory processes, myricetin can help downregulate post-ischemic neuroinflammation. This polyphenol acts as an inhibitor of the nucleotide-binding oligomerization domain-like receptor protein 3 (NLRP3). After its activation, this receptor forms an inflammasome that triggers caspase-1, leading to the maturation of pro-inflammatory cytokines such as IL-1β. Due to myricetin activity, these processes are suppressed, and therefore, inflammation can be halted [35,36].

Besides flavonoids, polyphenols include other families, such as stilbenes. The best-known stilbenoid is resveratrol. This compound can be found mainly in grapes [37,38]. Similar to other mentioned polyphenols, resveratrol acts as an anti-inflammatory, antioxidative, anticarcinogenic, cardioprotective, and immunomodulatory agent. The presence of hydroxyl groups and a double-bonded ethylene bridge is the cause of resveratrol’s instability and sensitivity to pH, light, and increased temperature [39,40]. Additionally, resveratrol is quickly metabolized and has poor solubility and a low rate of bioavailability (<10 ng/mL after 25 mg intake) [41,42].

Resveratrol is another polyphenol reported to have inhibitory effects on the NF-κB signaling pathway. The mechanism of resveratrol’s action on this pathway is based on influencing different targets. One of them may be the activation of sirtuin-1 (Sirt-1), which is a deacetylase that suppresses the TLR-4/NF-κB/STAT pathway. Additionally, resveratrol can prevent NF-κB activation mediated by the inhibitor of κBβ (IκBβ) and by IL-1β. In the case of the MAPK family, resveratrol can inhibit all of its kinases [43]. Lowering the expression of COX-2 and iNOS by resveratrol is another common pathway for polyphenols for decreasing inflammation [44]. Resveratrol can also downregulate inflammation by the inhibition of activator protein-1 (AP-1), which regulates gene expression related to inflammatory processes [43]. Resveratrol was also proven to lower the amount of pro-inflammatory cytokines such as TNF-α, IFN-γ, IL-2, IL-9, IL-12, and IL-17 [45].

Curcuminoids serve as another example of the polyphenol family. The most prevalent member of this group is curcumin, exhibiting anti-inflammatory, immunomodulatory, and antioxidant properties [46]. Curcumin has low bioavailability and absorbs poorly after oral intake; therefore, despite its broad range of potential applications, it is difficult to exploit its full potential. One of the ways of increasing curcumin’s bioavailability by 2000% is simultaneous consumption of piperine [47,48].

Curcumin’s anti-inflammatory effect can be seen, for instance, through its ability to lower monocyte chemoattractant/chemotactic protein-1 (MCP-1) expression. This chemokine intensifies the inflammatory response, and various studies have indicated that curcumin inhibits the synthesis of MCP-1. Curcumin can exert this effect, similarly to other polyphenols, by influencing several signaling pathways, mainly MAPK (especially JNK) and NF-κB but also protein kinase C (PKC) and MMP, which are considered targets [49]. Curcumin was also reported to exhibit anti-inflammatory properties by influencing the Janus kinase/signal transducer and activator of transcription (JAK/STAT) signaling pathway [50]. The inhibition of NF-κB by curcumin was additionally proven to lower the activity of COX-2, iNOS, and lipoxygenase (LOX) [49]. Another consequence of curcumin acting on NF-κB is suppressing the activation of the NLRP3 inflammasome [50,51].

To assess the effectiveness of the mentioned polyphenols in reducing inflammation, in our study, HBEC-5i cells were stimulated with TNF-α. This pro-inflammatory agent, under in vivo conditions, acts specifically on the brain endothelial cells by activating the TLR4/NFκB signaling pathway and phosphatidylinositol-3 kinase (PI3K), which results in lowered expression of TJs proteins (ZO-1, claudin-5, and occludin). As a consequence, the BBB is disrupted, which may be the initial step of neuroinflammation and neurodegeneration [5,10]. TNF-α also contributes to the disruption of the BBB by activating Rho-kinase and stimulating the production of substance P (SP) with its receptor neurokinin 1 (NK1R). RhoA protein, after binding with the Rho-kinase, causes phosphorylation of the myosin light chain (MLC), resulting in the disruption of TJs. Additionally, the level of nitric oxide (NO) acting as an antioxidant is lowered. SP, by acting on NK1R, induces the activation of the majority of cytokines and therefore takes part in the inflammatory processes [25].

TNF-α activates many signaling pathways in the brain endothelial cells, resulting in an increase in inflammation. This mechanism of action leads to the secretion of pro-inflammatory cytokines such as IL-6, IL-8, and IL-1β, which may serve as indicators of the ongoing inflammation.

One of the primary roles of IL-6 is to cause the differentiation and activation of B cells, which allows them to synthesize antibodies. IL-6 also fulfils the role of activating T lymphocytes and causes an increase in their number [52]. This cytokine appears rapidly after infection or injury. The intensified production of IL-6 leads to chronic inflammation, which is the cause of many pathological conditions [53,54]. The secretion of IL-6 after stimulation of the brain endothelial cells with TNF-α was observed in models of neuroinflammation, including the study conducted by Rochfort et al. and our research [55]. The elevated IL-6 level in HBEC-5i cells cultured in medium alone, compared with the control group, indicates the correct induction of the inflammatory response. The polyphenols assessed for anti-inflammatory properties demonstrated effectiveness. The application of chrysin and myricetin was confirmed, with statistical significance, to attenuate inflammation by lowering IL-6 levels. Our findings regarding chrysin align with the results of a study by Ma et al. performed on human coronary artery endothelial cells. The cells stimulated with TNF-α secreted an increased level of IL-6, which was downregulated by chrysin. The proposed chrysin’s mechanism of action relies on modulation of nuclear factor of activated T cells 2 (NFAT2) [56]. Similarly, the study on human umbilical vein endothelial cells (HUVECs), induced with lipopolysaccharide (LPS), conducted by Lin et al., revealed that chrysin decreases the IL-6 concentration [57]. In the case of myricetin, its effectiveness was also confirmed by Chen et al. in a study on A549 cells. The upregulated IL-6 level after TNF-α stimulation was reduced by myricetin. Myricetin, by activating Sirt-1, leads to the reduction of NF-κB p65 and p53 acetylation, which results in the inhibition of IL-6 secretion [58]. In the case of resveratrol and curcumin, their anti-inflammatory effect was visible, but it cannot be confirmed statistically that these polyphenols are effective agents against inflammation in the HBEC-5i cell environment. Despite our findings, a study conducted by Kolahdouz-Mohammadi et al. reports that resveratrol effectively downregulates IL-6 expression in endometrial stromal cells from patients with endometriosis [59]. Regarding curcumin’s ability to reduce IL-6 concentration, it was confirmed by Cho et al. in a study on HaCaT cells induced with TNF-α. Curcumin inhibited the activation of MAPKs and NF-κB, leading to the downregulation of IL-6 expression [60].

IL-8 is another tested cytokine, secreted in response to tissue injury and infection. It exhibits pro-inflammatory properties by recruiting lymphocytes, especially neutrophils, to the target area [61,62]. Additionally, IL-8 activates neutrophils, allowing them to discharge their granule contents [63]. In our research, it was observed that the stimulation of HBEC-5i cells with TNF-α induced strong secretion of IL-8 in comparison to the control group. This response of brain endothelial cells to TNF-α was also confirmed in the study conducted by O’Carroll et al. [64]. The anti-inflammatory activity of polyphenols was similar to that previously described. Chrysin and myricetin significantly decreased the concentration of the pro-inflammatory cytokine IL-8, whereas resveratrol and curcumin showed only a tendency to lower inflammation, without statistical confirmation. The activity of chrysin in reducing the expression of IL-8 was also confirmed in a study by Lee et al. conducted on HEK 293 cells stimulated with TNF-α. The results indicated that chrysin suppresses IL-8 promoter activation and gene expression. Additionally, chrysin prevents the translocation of NF-κB p65 [65]. Regarding myricetin, Chen et al. obtained similar results in a previously mentioned study on A549 cells. The elevated IL-8 level induced by TNF-α was reduced by myricetin [58]. Contrary to our findings regarding resveratrol, Oh et al. demonstrated that it can decrease IL-8 production in LPS-induced THP-1 cells. It was concluded that resveratrol achieves this effect by inhibiting MAPK phosphorylation and NF-κB activation [66]. In the context of curcumin’s effectiveness, Wang et al. demonstrated in their study on HUVECs that this polyphenol decreases the expression of IL-8 by modulating the ERK and p38 MAPK signaling pathways [67].

We demonstrated in our study that stimulation of HBEC-5i cells with TNF-α results in the secretion of another pro-inflammatory cytokine, which is IL-1β. This cytokine initially exists in an inactive form, pro-IL-1β. In its activation to the mature form, the NLRP3 inflammasome is involved. IL-1β is released by activated macrophages under inflammatory conditions and is engaged in the attraction of leukocytes to the affected site [68]. Additionally, it is involved in the activation and differentiation of T lymphocytes, which produce IL-17 cytokine [69]. Similar to the previous results, chrysin and myricetin effectively suppressed the production of IL-1β cytokine, proving their anti-inflammatory properties statistically. Similar results regarding chrysin were reported by Ha et al. in a study on microglia activated with LPS. Chrysin was shown to decrease IL-1β secretion. Additionally, it was confirmed that this polyphenol suppresses the activation of JNK and NF-κB [27]. The ability of myricetin to lower the expression of IL-1β was confirmed in a study conducted by Park et al. on RAW 264.7 murine macrophages. The cells treated with endocrine-disrupting chemical Di(2-ethylhexyl) phthalate (DEHP) showed an increased level of IL-1β, which was reduced by myricetin, acting as an inhibitor of PKC and ERK [70]. Resveratrol and curcumin use were associated with the reduction of IL-1β; however, these changes were not statistically significant. Despite our findings concerning resveratrol, the study by Tian et al. on human rheumatoid arthritis fibroblast-like synoviocytes induced with TNF-α revealed that it can reduce the amount of IL-1β by inhibiting the PI3K-Akt signaling pathway [71]. Regarding curcumin, the study confirming its ability to decrease IL-1β levels was conducted by Yin et al. in mouse bone marrow–derived macrophages induced with LPS. The proposed curcumin’s mechanism of action is based on inhibition of NLRP3 inflammasome activation [72].

The effectiveness of tested polyphenols in reducing the amount of the pro-inflammatory cytokines IL-6, IL-8, and IL-1β can be attributed to their ability to inhibit several signaling pathways responsible for the increase of inflammation. Chrysin, myricetin, resveratrol, and curcumin have been shown to suppress the activity of the NF-κB and MAPK pathways, which are the primary inflammatory regulators responsible for the secretion of the mentioned cytokines. The limited effectiveness of resveratrol and curcumin in the inhibition of cytokine production by HBEC-5i cells may be attributed to their especially low stability. These data lay the foundations for further research focusing on the enhancement of their durability and delivery methods. Chrysin and myricetin proved their efficacy; however, myricetin emerged as the optimal candidate for downregulating inflammation in the HBEC-5i cell environment since it is most effective at the lowest concentration.

We also focused in our research on measuring the expression of vascular cell adhesion molecule-1 (*VCAM-1*) present on the surface of brain endothelial cells after stimulation with TNF-α. *VCAM-1* belongs to the group of cell adhesion molecules, present mainly on endothelial cells, which under inflammatory conditions are responsible for adhesion of leukocytes to the cell surface and allow for their transendothelial migration. The process of leukocyte adhesion to *VCAM-1* is possible due to the presence of α4β1 integrin on the leukocyte surface, which directly binds to *VCAM-1*, leading to the activation of signaling pathways and resulting in TJs deterioration and remodeling of actin [73,74]. Similarly to the results obtained in the study conducted by O’Carroll et al., we observed intensified expression of *VCAM-1* after the treatment of HBEC-5i cells with TNF-α [64]. Also, the research conducted by Wojkowska and Szpakowski on the murine brain endothelial cells revealed that their induction with TNF-α results in the increase of *VCAM-1* presence [74]. The expression of *VCAM-1* after stimulation with TNF-α is possible because this cytokine induces signaling pathways in which NF-κB and activator protein 1 (AP1), belonging to the MAPK cascade, are activated and stimulate the expression of the *VCAM-1* gene [73,75,76]. The increased level of *VCAM-1* favors inflammation; therefore, it is essential to identify the anti-inflammatory agents able to lower its expression. Our research shows that the tested polyphenols turned out to be the optimal candidates. Chrysin and resveratrol decreased the amount of *VCAM-1* on the surface of HBEC-5i cells compared with the TNF-α-only group, validating their effectiveness in eradicating inflammation. Our results regarding chrysin’s effectiveness in attenuating *VCAM-1* expression in brain endothelial cells are consistent with the study by Lee et al. on murine brain endothelial cells. This study demonstrated that chrysin reduces the expression of *VCAM-1* by suppressing NF-κB translocation and the MAPK pathway [77]. The study conducted by Zhao et al. also confirms chrysin’s mechanism of action [78]. The findings from our study focusing on resveratrol’s ability to downregulate *VCAM-1* expression align with the study by Zhang et al., where endothelial progenitor cells were treated with TNF-α and resveratrol. Similarly to our results, TNF-α upregulated the expression of *VCAM-1*, while application of resveratrol led to its reduction by NF-κB inhibition [79]. Myricetin and curcumin also reduced the expression of *VCAM-1*; however, their activity was not statistically significant when compared with more effective resveratrol and chrysin. However, in the literature, studies can be found confirming the effectiveness of these polyphenols on different types of cells. Bai et al. report that myricetin downregulates *VCAM-1* expression in HUVECs induced with oxidized low-density lipoprotein [80]. In the case of curcumin, Kim et al. confirm in the study on human endometriotic stromal cells induced with TNF-α that this polyphenol reduces the expression of *VCAM-1* [81]. Because of the involvement of NF-κB and MAPK in *VCAM-1* overexpression and the proven ability of the tested polyphenols to inhibit activities of these signaling pathways, it can be concluded that these pathways constitute the primary targets of chrysin, myricetin, curcumin, and resveratrol.

Polyphenols are known for their antioxidant properties, which we also confirmed in our study on HBEC-5i cells. Their ability to eliminate ROS is mainly based on the abundant presence of phenolic hydroxyl groups acting as active hydrogen donors. ROS, after receiving the hydrogen atom, becomes a stable molecule. Polyphenols, after losing a hydrogen atom, are safe for cells and less toxic than ROS. Additionally, polyphenols increase the activity of antioxidant enzymes [82]. In our study, only curcumin lacked antioxidant activity. This observation can be explained by the fact that curcumin is highly unstable. Under conditions of 37 °C and pH 7.2, the t_1/2_ for curcumin is approximately 10 min. Additionally, curcumin can contribute to the generation of ROS [48]. This polyphenol can covalently modify Cys496 and Sec497 residues in thioredoxin reductase (TrxR), which leads to the loss of its reduction activity of thioredoxins (Trxs) and transformation into NADPH oxidase, which produces ROS [83]. The selected polyphenols in our study also showed potency in increasing the viability of HBEC-5i cells. The mechanism of action behind this phenomenon is based on the polyphenols’ influence on mitochondria. The tested polyphenols activate Sirt-1, leading to the deacetylation of PGC-1α. In consequence, the transcription of nuclear and mitochondrial genes takes place, resulting in the increased proliferation of mitochondria and therefore improved cell metabolism and survival [84,85,86,87].

## 4. Materials and Methods

### 4.1. Cerebral Microvascular Endothelial Cell Culture

The cerebral microvascular endothelial cell line (HBEC-5i) was isolated from human cerebral cortex and purchased from ATCC (Manassas, VA, USA; CRL-3245™). The cells were cultured in 75 cm^2^ culture flasks coated with 0.1% gelatin (Sigma–Aldrich, St. Louis, MO, USA, 1 mL per 10 cm^2^. Cells were grown in culture medium DMEM-F12 (Biowest, Nuaillé, France) supplemented with 1% of Antibiotic Antimycotic Solution (penicillin, streptomycin, amphotericin) (Sigma–Aldrich), 40 μg/mL endothelial growth supplement (ECGS) (Corning^®^, Corning, NY, USA), and 10% of heat-inactivated fetal bovine serum (FBS) (Biowest, Nuaillé, France) in 5% CO_2_ atmosphere, 37 °C, and increased humidity. The culture medium was renewed every 2–3 days. When the cell culture reached 90% confluency, the cells were washed with Dulbecco’s PBS (Biowest, Nuaillé, France) and then collected with the use of Trypsin-EDTA (ethylenediaminetetraacetic acid) solution (Sigma–Aldrich) and 10% FBS in PBS, centrifuged (125× *g*, 6 min, 20 °C), and seeded on 24-well culture plates coated with gelatin (70 × 10^3^/well), and cultured for 24 h, enabling cells to bind to the growth surface.

### 4.2. Stimulation of HBEC-5i Cells with TNF-α and Polyphenols

HBEC-5i cells were stimulated with 5 ng/mL rhTNF-α (R&D Systems, Minneapolis, MN, USA) to induce the pro-inflammatory response and also with polyphenols: chrysin, myricetin, curcumin, and resveratrol (all reagents from Sigma–Aldrich), at concentrations of 1 nM for myricetin and 10 nM for the rest, to confirm their anti-inflammatory properties and establish the most effective concentration. The control group constituted cells cultured in medium alone. The cells after the stimulation were incubated for 48 h in 24-well culture plates. After this time, the culture medium was collected and centrifuged (5000× *g*, 10 min, 20 °C). The obtained supernatants were placed at −80 °C. Simultaneously with the medium collection, the remaining cells were harvested after 48 h and prepared for flow cytometry analysis.

### 4.3. Analysis of HBEC-5i Cell Response with the ELISA Test

The concentrations of cytokines secreted by HBEC-5i cells were measured using ELISA kits (DuoSet, R&D Systems, Minneapolis, MN, USA) according to the manufacturer’s protocols. The collected culture media were analyzed in terms of the presence of the pro-inflammatory cytokines IL-1ß, IL-6, and IL-8. The supernatants used for the measurement of IL-6 and IL-8 concentrations were diluted 50 times prior to the ELISA test. The concentration of IL-1ß was measured without dilution of culture media.

### 4.4. Assessment of CD106 (VCAM-1) Expression with Flow Cytometry

After 48 h of incubation, HBEC-5i cells were collected on ice using cell dissociation solution (Sigma–Aldrich, St. Louis, MO, USA). Next, the cells, including antibody isotype controls, were washed with cold 1% FBS in PBS and stained with anti-CD106 PE mouse IgG1 κ (Biolegend, San Diego, CA, USA). The cells were incubated with the fluorescently labelled antibodies for 30 min, 4 °C, in the dark, then washed twice with cold 1% FBS in PBS, fixed with formalin (20 min, on ice), again washed twice with cold 1% FBS in PBS, and frozen in FBS with 10% dimethyl sulfoxide (DMSO; Sigma–Aldrich, St. Louis, MO, USA). Flow cytometry was performed on an LSR II flow cytometer (Becton Dickinson, San Jose, CA, USA).

### 4.5. Measurement of HBEC-5i Cell Viability and Antioxidative Properties of Polyphenols

HBEC-5i cells were seeded into two 96-well plates at a density of 12.5 × 10^3^/well under 5% CO_2_, 37 °C conditions. After 24 h, the cells on both plates were stimulated with 1 nM mirycetin, 10 nM chrysin, 10 nM resveratrol, and 10 nM curcumin, excluding the control group. After 24 h of stimulation with polyphenols, cells from one of the plates were treated with 250 µL H_2_O_2_; the other plate was incubated without changes. Then, 48 h after stimulation with polyphenols, the viability and antioxidative properties of the polyphenols were measured using EZMTT assay (Merck Millipore, Darmstadt, Germany). After incubation, the medium was removed and replaced with 100 µL of fresh medium containing EZMTT reagent diluted 200 times per well. The plates were incubated for 3 h at 37 °C, and the absorbance was measured at λ = 450 nm.

### 4.6. Statistical Analysis

For comparisons between two groups, the non-parametric Mann–Whitney U test was conducted. Comparisons between multiple groups were performed using non-parametric ANOVA. The non-parametric analysis of the data was performed due to the low sample number. When Kruskal–Wallis tests were considered significant (*p* < 0.05), post-hoc Dunn’s tests were utilized for precise comparisons between groups. Statistical tests were performed using Statistica 13.00. Flow cytometry data analysis was performed using FlowJo 10 software.

## 5. Conclusions

Brain endothelial cells present themselves as a promising target for anti-inflammatory agents such as polyphenols. In our study, myricetin, chrysin, resveratrol, and curcumin were tested for decreasing the inflammatory response of HBEC-5i cells after stimulation with pro-inflammatory TNF-α. Myricetin and chrysin showed statistically confirmed effectiveness in lowering the concentration of secreted IL-6, IL-8, and IL-1β. Additionally, myricetin was the most effective at the lowest concentration. Resveratrol and curcumin presented a tendency in decreasing inflammation; however, these results were not statistically significant. Chrysin and resveratrol reduced the expression of *VCAM-1*, which is a molecule common for endothelial cells, responsible for intensifying inflammation. The selected polyphenols also increased the viability of HBEC-5i cells and showed antioxidant properties. The effects of polyphenols presented here suggest that they are optimal candidates for combating inflammation in the BBB environment. Despite promising results in the in vitro study, they might not be as effective in vivo. Due to their poor bioavailability, solubility, and high rate of metabolism, it is essential to develop strategies allowing for the delivery of polyphenols to the target, where their anti-inflammatory properties can be utilized.

## Data Availability

The raw data supporting the conclusions of this article will be made available by the authors on request.

## References

[1] Wang Y., Wang N., Cai B., Wang G., Li J., Piao X. (2015). In Vitro Model of the Blood-Brain Barrier Established by Co-Culture of Primary Cerebral Microvascular Endothelial and Astrocyte Cells. Neural Regen. Res..

[2] Li X., Simo L., Zhao Q., Kim E.G., Ding Y., Geng X. (2025). Endothelial Cells and the Blood–Brain Barrier: Critical Determinants of Ineffective Reperfusion in Stroke. Eur. J. Neurosci..

[3] Grammas P., Martinez J., Miller B. (2011). Cerebral Microvascular Endothelium and the Pathogenesis of Neurodegenerative Diseases. Expert. Rev. Mol. Med..

[4] McConnell H.L., Mishra A. (2022). Cells of the Blood–Brain Barrier: An Overview of the Neurovascular Unit in Health and Disease. Methods Mol. Biol..

[5] Xiang Y., Gu Q., Liu D. (2025). Brain Endothelial Cells in Blood–Brain Barrier Regulation and Neurological Therapy. Int. J. Mol. Sci..

[6] Chow B.W., Gu C. (2015). The Molecular Constituents of the Blood–Brain Barrier. Trends Neurosci..

[7] Ashby J.W., Mack J.J. (2021). Endothelial Control of Cerebral Blood Flow. Am. J. Pathol..

[8] Patabendige A., Janigro D. (2023). The Role of the Blood–Brain Barrier during Neurological Disease and Infection. Biochem. Soc. Trans..

[9] Correale J., Gaitán M.I., Ysrraelit M.C., Fiol M.P. (2017). Progressive Multiple Sclerosis: From Pathogenic Mechanisms to Treatment. Brain.

[10] Takata F., Nakagawa S., Matsumoto J., Dohgu S. (2021). Blood-Brain Barrier Dysfunction Amplifies the Development of Neuroinflammation: Understanding of Cellular Events in Brain Microvascular Endothelial Cells for Prevention and Treatment of BBB Dysfunction. Front. Cell. Neurosci..

[11] Zierfuss B., Larochelle C., Prat A. (2024). Blood–Brain Barrier Dysfunction in Multiple Sclerosis: Causes, Consequences, and Potential Effects of Therapies. Lancet Neurol..

[12] Fellner L., Irschick R., Schanda K., Reindl M., Klimaschewski L., Poewe W., Wenning G.K., Stefanova N. (2013). Toll-like Receptor 4 Is Required for A-synuclein Dependent Activation of Microglia and Astroglia. Glia.

[13] Ranaivo H.R., Hodge J.N., Choi N., Wainwright M.S. (2012). Albumin Induces Upregulation of Matrix Metalloproteinase-9 in Astrocytes via MAPK and Reactive Oxygen Species-Dependent Pathways. J. Neuroinflamm..

[14] Yu Q., Tao H., Wang X., Li M. (2015). Targeting Brain Microvascular Endothelial Cells: A Therapeutic Approach to Neuroprotection against Stroke. Neural Regen. Res..

[15] Fraga C.G., Croft K.D., Kennedy D.O., Tomás-Barberán F.A. (2019). The Effects of Polyphenols and Other Bioactives on Human Health. Food Funct..

[16] Abbas M., Saeed F., Anjum F.M., Afzaal M., Tufail T., Bashir M.S., Ishtiaq A., Hussain S., Suleria H.A.R. (2017). Natural Polyphenols: An Overview. Int. J. Food Prop..

[17] Hussain T., Tan B., Yin Y., Blachier F., Tossou M.C.B., Rahu N. (2016). Oxidative Stress and Inflammation: What Polyphenols Can Do for Us?. Oxid. Med. Cell. Longev..

[18] Rudrapal M., Rakshit G., Singh R.P., Garse S., Khan J., Chakraborty S. (2024). Dietary Polyphenols: Review on Chemistry/Sources, Bioavailability/Metabolism, Antioxidant Effects, and Their Role in Disease Management. Antioxidants.

[19] Rudrapal M., Khairnar S.J., Khan J., Bin Dukhyil A., Ansari M.A., Alomary M.N., Alshabrmi F.M., Palai S., Deb P.K., Devi R. (2022). Dietary Polyphenols and Their Role in Oxidative Stress-Induced Human Diseases: Insights Into Protective Effects, Antioxidant Potentials and Mechanism(s) of Action. Front. Pharmacol..

[20] Yahfoufi N., Alsadi N., Jambi M., Matar C. (2018). The Immunomodulatory and Anti-Inflammatory Role of Polyphenols. Nutrients.

[21] Jantan I., Haque A., Arshad L., Harikrishnan H., Septama A.W., Mohamed-Hussein Z.-A. (2021). Dietary Polyphenols Suppress Chronic Inflammation by Modulation of Multiple Inflammation-Associated Cell Signaling Pathways. J. Nutr. Biochem..

[22] Mani R., Natesan V. (2018). Chrysin: Sources, Beneficial Pharmacological Activities, and Molecular Mechanism of Action. Phytochemistry.

[23] Mishra A., Mishra P.S., Bandopadhyay R., Khurana N., Angelopoulou E., Paudel Y.N., Piperi C. (2021). Neuroprotective Potential of Chrysin: Mechanistic Insights and Therapeutic Potential for Neurological Disorders. Molecules.

[24] Stompor-Gorący M., Bajek-Bil A., Machaczka M. (2021). Chrysin: Perspectives on Contemporary Status and Future Possibilities as Pro-Health Agent. Nutrients.

[25] Gao X., Bayraktutan U. (2023). TNF-α Evokes Blood-Brain Barrier Dysfunction through Activation of Rho-Kinase and Neurokinin 1 Receptor. Immunobiology.

[26] Zeinali M., Rezaee S.A., Hosseinzadeh H. (2017). An Overview on Immunoregulatory and Anti-Inflammatory Properties of Chrysin and Flavonoids Substances. Biomed. Pharmacother..

[27] Ha S.K., Moon E., Kim S.Y. (2010). Chrysin Suppresses LPS-Stimulated Proinflammatory Responses by Blocking NF-ΚB and JNK Activations in Microglia Cells. Neurosci. Lett..

[28] Agraharam G., Girigoswami A., Girigoswami K. (2022). Myricetin: A Multifunctional Flavonol in Biomedicine. Curr. Pharmacol. Rep..

[29] Semwal D., Semwal R., Combrinck S., Viljoen A. (2016). Myricetin: A Dietary Molecule with Diverse Biological Activities. Nutrients.

[30] Imran M., Saeed F., Hussain G., Imran A., Mehmood Z., Gondal T.A., El-Ghorab A., Ahmad I., Pezzani R., Arshad M.U. (2021). Myricetin: A Comprehensive Review on Its Biological Potentials. Food Sci. Nutr..

[31] Taheri Y., Suleria H.A.R., Martins N., Sytar O., Beyatli A., Yeskaliyeva B., Seitimova G., Salehi B., Semwal P., Painuli S. (2020). Myricetin Bioactive Effects: Moving from Preclinical Evidence to Potential Clinical Applications. BMC Complement. Med. Ther..

[32] Jang J.-H., Lee S.H., Jung K., Yoo H., Park G. (2020). Inhibitory Effects of Myricetin on Lipopolysaccharide-Induced Neuroinflammation. Brain Sci..

[33] Rahmani A., Almatroudi A., Allemailem K., Alwanian W., Alharbi B., Alrumaihi F., Khan A., Almatroodi S. (2023). Myricetin: A Significant Emphasis on Its Anticancer Potential via the Modulation of Inflammation and Signal Transduction Pathways. Int. J. Mol. Sci..

[34] Song X., Tan L., Wang M., Ren C., Guo C., Yang B., Ren Y., Cao Z., Li Y., Pei J. (2021). Myricetin: A Review of the Most Recent Research. Biomed. Pharmacother..

[35] Pluta R., Januszewski S., Czuczwar S.J. (2021). Myricetin as a Promising Molecule for the Treatment of Post-Ischemic Brain Neurodegeneration. Nutrients.

[36] Lu Y., Wang T., Yu B., Xia K., Guo J., Liu Y., Ma X., Zhang L., Zou J., Chen Z. (2025). Mechanism of Action of the Nucleotide-Binding Oligomerization Domain-like Receptor Protein 3 Inflammasome and Its Regulation in Liver Injury. Chin. Med. J..

[37] Hasan M., Bae H. (2017). An Overview of Stress-Induced Resveratrol Synthesis in Grapes: Perspectives for Resveratrol-Enriched Grape Products. Molecules.

[38] Zhang L.-X., Li C.-X., Kakar M.U., Khan M.S., Wu P.-F., Amir R.M., Dai D.-F., Naveed M., Li Q.-Y., Saeed M. (2021). Resveratrol (RV): A Pharmacological Review and Call for Further Research. Biomed. Pharmacother..

[39] Tian B., Liu J. (2020). Resveratrol: A Review of Plant Sources, Synthesis, Stability, Modification and Food Application. J. Sci. Food Agric..

[40] Chan E.W.C., Wong C.W., Tan Y.H., Foo J.P.Y., Wong S.K., Chan H.T. (2019). Resveratrol and Pterostilbene: A Comparative Overview of Their Chemistry, Biosynthesis, Plant Sources and Pharmacological Properties. J. Appl. Pharm. Sci..

[41] Pannu N., Bhatnagar A. (2019). Resveratrol: From Enhanced Biosynthesis and Bioavailability to Multitargeting Chronic Diseases. Biomed. Pharmacother..

[42] Diaz-Gerevini G.T., Repossi G., Dain A., Tarres M.C., Das U.N., Eynard A.R. (2016). Beneficial Action of Resveratrol: How and Why?. Nutrition.

[43] Meng T., Xiao D., Muhammed A., Deng J., Chen L., He J. (2021). Anti-Inflammatory Action and Mechanisms of Resveratrol. Molecules.

[44] De Sá Coutinho D., Pacheco M.T., Frozza R.L., Bernardi A. (2018). Anti-Inflammatory Effects of Resveratrol: Mechanistic Insights. Int. J. Mol. Sci..

[45] Repossi G., Das U.N., Eynard A.R. (2020). Molecular Basis of the Beneficial Actions of Resveratrol. Arch. Med. Res..

[46] Kotha R.R., Luthria D.L. (2019). Curcumin: Biological, Pharmaceutical, Nutraceutical, and Analytical Aspects. Molecules.

[47] Heidari H., Bagherniya M., Majeed M., Sathyapalan T., Jamialahmadi T., Sahebkar A. (2023). Curcumin-piperine Co-supplementation and Human Health: A Comprehensive Review of Preclinical and Clinical Studies. Phytother. Res..

[48] Nelson K.M., Dahlin J.L., Bisson J., Graham J., Pauli G.F., Walters M.A. (2017). The Essential Medicinal Chemistry of Curcumin. J. Med. Chem..

[49] Sadeghi M., Dehnavi S., Asadirad A., Xu S., Majeed M., Jamialahmadi T., Johnston T.P., Sahebkar A. (2023). Curcumin and Chemokines: Mechanism of Action and Therapeutic Potential in Inflammatory Diseases. Inflammopharmacology.

[50] Peng Y., Ao M., Dong B., Jiang Y., Yu L., Chen Z., Hu C., Xu R. (2021). Anti-Inflammatory Effects of Curcumin in the Inflammatory Diseases: Status, Limitations and Countermeasures. Drug Des. Devel. Ther..

[51] Varì R., Scazzocchio B., Silenzi A., Giovannini C., Masella R. (2021). Obesity-Associated Inflammation: Does Curcumin Exert a Beneficial Role?. Nutrients.

[52] Aliyu M., Zohora F.T., Anka A.U., Ali K., Maleknia S., Saffarioun M., Azizi G. (2022). Interleukin-6 Cytokine: An Overview of the Immune Regulation, Immune Dysregulation, and Therapeutic Approach. Int. Immunopharmacol..

[53] Roohi E., Jaafari N., Hashemian F. (2021). On Inflammatory Hypothesis of Depression: What Is the Role of IL-6 in the Middle of the Chaos?. J. Neuroinflamm..

[54] Jordan S.C., Choi J., Kim I., Wu G., Toyoda M., Shin B., Vo A. (2017). Interleukin-6, A Cytokine Critical to Mediation of Inflammation, Autoimmunity and Allograft Rejection. Transplantation.

[55] Rochfort K.D., Collins L.E., McLoughlin A., Cummins P.M. (2016). Tumour Necrosis Factor-α-mediated Disruption of Cerebrovascular Endothelial Barrier Integrity in Vitro Involves the Production of Proinflammatory Interleukin-6. J. Neurochem..

[56] Ma J., Li Y., Tang Y., Qian G., Lv H., Song X., Liu Y. (2025). Chrysin Improves Endothelial Inflammation via the NFAT Pathway in Kawasaki Disease. Mol. Biol. Rep..

[57] Lin C.-M., Chang H., Li S.-Y., Wu I.-H., Chiu J.-H. (2006). Chrysin Inhibits Lipopolysaccharide-Induced Angiogenesis via Down-Regulation of VEGF/VEGFR-2(KDR) and IL-6/IL-6R Pathways. Planta Med..

[58] Chen M., Chen Z., Huang D., Sun C., Xie J., Chen T., Zhao X., Huang Y., Li D., Wu B. (2020). Myricetin Inhibits TNF-α-Induced Inflammation in A549 Cells via the SIRT1/NF-ΚB Pathway. Pulm. Pharmacol. Ther..

[59] Kolahdouz-Mohammadi R., Shidfar F., Khodaverdi S., Arablou T., Heidari S., Rashidi N., Delbandi A. (2021). Resveratrol Treatment Reduces Expression of MCP-1, IL-6, IL-8 and RANTES in Endometriotic Stromal Cells. J. Cell. Mol. Med..

[60] Cho J.-W., Lee K.-S., Kim C.-W. (2007). Curcumin Attenuates the Expression of IL-1β, IL-6, and TNF-α as Well as Cyclin E in TNF-α-Treated HaCaT Cells; NF-ΚB and MAPKs as Potential Upstream Targets. Int. J. Mol. Med..

[61] David J., Dominguez C., Hamilton D., Palena C. (2016). The IL-8/IL-8R Axis: A Double Agent in Tumor Immune Resistance. Vaccines.

[62] Long X., Ye Y., Zhang L., Liu P., Yu W., Wei F., Ren X., Yu J. (2016). IL-8, a Novel Messenger to Cross-Link Inflammation and Tumor EMT via Autocrine and Paracrine Pathways. Int. J. Oncol..

[63] Matsushima K., Yang D., Oppenheim J.J. (2022). Interleukin-8: An Evolving Chemokine. Cytokine.

[64] O’Carroll S.J., Kho D.T., Wiltshire R., Nelson V., Rotimi O., Johnson R., Angel C.E., Graham E.S. (2015). Pro-Inflammatory TNFα and IL-1β Differentially Regulate the Inflammatory Phenotype of Brain Microvascular Endothelial Cells. J. Neuroinflamm..

[65] Lee S.-H., Kim Y.-J., Kwon S.-H., Lee Y.-H., Choi S.-Y., Park J.-S., Kwon H.-J. (2009). Inhibitory Effects of Flavonoids on TNF-α-Induced IL-8 Gene Expression in HEK 293 Cells. BMB Rep..

[66] Oh Y.-C., Kang O.-H., Choi J.-G., Chae H.-S., Lee Y.-S., Brice O.-O., Jung H.J., Hong S.-H., Lee Y.-M., Kwon D.-Y. (2009). Anti-Inflammatory Effect of Resveratrol by Inhibition of IL-8 Production in LPS-Induced THP-1 Cells. Am. J. Chin. Med..

[67] Wang J., Dong S. (2012). ICAM-1 and IL-8 Are Expressed by DEHP and Suppressed by Curcumin Through ERK and P38 MAPK in Human Umbilical Vein Endothelial Cells. Inflammation.

[68] Katkenov N., Mukhatayev Z., Kozhakhmetov S., Sailybayeva A., Bekbossynova M., Kushugulova A. (2024). Systematic Review on the Role of IL-6 and IL-1β in Cardiovascular Diseases. J. Cardiovasc. Dev. Dis..

[69] Cai Y., Xue F., Quan C., Qu M., Liu N., Zhang Y., Fleming C., Hu X., Zhang H., Weichselbaum R. (2019). A Critical Role of the IL-1β–IL-1R Signaling Pathway in Skin Inflammation and Psoriasis Pathogenesis. J. Investig. Dermatol..

[70] Park J.-Y., Lee S.-J. (2025). Myricetin Alleviates the Mechanism of IL-1β Production Caused by the Endocrine-Disrupting Chemical Di(2-Ethylhexyl) Phthalate in RAW 264.7 Cells. Tissue Cell.

[71] Tian J., Chen J., Gao J., Li L., Xie X. (2013). Resveratrol Inhibits TNF-α-Induced IL-1β, MMP-3 Production in Human Rheumatoid Arthritis Fibroblast-like Synoviocytes via Modulation of PI3kinase/Akt Pathway. Rheumatol. Int..

[72] Yin H., Guo Q., Li X., Tang T., Li C., Wang H., Sun Y., Feng Q., Ma C., Gao C. (2018). Curcumin Suppresses IL-1β Secretion and Prevents Inflammation through Inhibition of the NLRP3 Inflammasome. J. Immunol..

[73] Kong D.-H., Kim Y., Kim M., Jang J., Lee S. (2018). Emerging Roles of Vascular Cell Adhesion Molecule-1 (VCAM-1) in Immunological Disorders and Cancer. Int. J. Mol. Sci..

[74] Wojkowska D., Szpakowski P., Glabinski A. (2017). Interleukin 17A Promotes Lymphocytes Adhesion and Induces CCL2 and CXCL1 Release from Brain Endothelial Cells. Int. J. Mol. Sci..

[75] Wu S., Xu H., Peng J., Wang C., Jin Y., Liu K., Sun H., Qin J. (2015). Potent Anti-Inflammatory Effect of Dioscin Mediated by Suppression of TNF-α-Induced VCAM-1, ICAM-1and EL Expression via the NF-ΚB Pathway. Biochimie.

[76] Chen T., Zhang X., Zhu G., Liu H., Chen J., Wang Y., He X. (2020). Quercetin Inhibits TNF-α Induced HUVECs Apoptosis and Inflammation via Downregulating NF-KB and AP-1 Signaling Pathway in Vitro. Medicine.

[77] Lee B., Lee W., Jung Y.-S. (2017). Chrysin Attenuates VCAM-1 Expression and Monocyte Adhesion in Lipopolysaccharide-Stimulated Brain Endothelial Cells by Preventing NF-ΚB Signaling. Int. J. Mol. Sci..

[78] Zhao S., Liang M., Wang Y., Hu J., Zhong Y., Li J., Huang K., Li Y. (2019). Chrysin Suppresses Vascular Endothelial Inflammation via Inhibiting the NF-ΚB Signaling Pathway. J. Cardiovasc. Pharmacol. Ther..

[79] Zhang Y., Liu H., Tang W., Qiu Q., Peng J. (2020). Resveratrol Prevents TNF-α-Induced VCAM-1 and ICAM-1 Upregulation in Endothelial Progenitor Cells via Reduction of NF-ΚB Activation. J. Int. Med. Res..

[80] Bai Y., Liu X., Chen Q., Chen T., Jiang N., Guo Z. (2021). Myricetin Ameliorates Ox-LDL-Induced HUVECs Apoptosis and Inflammation via LncRNA GAS5 Upregulating the Expression of MiR-29a-3p. Sci. Rep..

[81] Kim K., Lee E.N., Park J.K., Lee J., Kim J., Choi H., Kim B., Lee H., Lee K., Yoon S. (2012). Curcumin Attenuates TNF-α-induced Expression of Intercellular Adhesion Molecule-1, Vascular Cell Adhesion Molecule-1 and Proinflammatory Cytokines in Human Endometriotic Stromal Cells. Phytother. Res..

[82] Lv Q., Long J., Gong Z., Nong K., Liang X., Qin T., Huang W., Yang L. (2021). Current State of Knowledge on the Antioxidant Effects and Mechanisms of Action of Polyphenolic Compounds. Nat. Prod. Commun..

[83] Fang J., Lu J., Holmgren A. (2005). Thioredoxin Reductase Is Irreversibly Modified by Curcumin. J. Biol. Chem..

[84] Chodari L., Dilsiz Aytemir M., Vahedi P., Alipour M., Vahed S.Z., Khatibi S.M.H., Ahmadian E., Ardalan M., Eftekhari A. (2021). Targeting Mitochondrial Biogenesis with Polyphenol Compounds. Oxid. Med. Cell. Longev..

[85] Yang Z., Guan Y., Li J., Li L., Li Z. (2018). Chrysin Attenuates Carrageenan-Induced Pleurisy and Lung Injury via Activation of SIRT1/NRF2 Pathway in Rats. Eur. J. Pharmacol..

[86] Jung H.-Y., Lee D., Ryu H.G., Choi B.-H., Go Y., Lee N., Lee D., Son H.G., Jeon J., Kim S.-H. (2017). Myricetin Improves Endurance Capacity and Mitochondrial Density by Activating SIRT1 and PGC-1α. Sci. Rep..

[87] Yang Y., Duan W., Lin Y., Yi W., Liang Z., Yan J., Wang N., Deng C., Zhang S., Li Y. (2013). SIRT1 Activation by Curcumin Pretreatment Attenuates Mitochondrial Oxidative Damage Induced by Myocardial Ischemia Reperfusion Injury. Free Radic. Biol. Med..

